# Number of the Medical Profession in Great Britain

**Published:** 1844-12

**Authors:** 


*Number of the Medical Profession in Great Britain.*—From the answers and returns made pursuant to an act, entitled, "An act for taking an account of the population of Great Britain," just published, it appears that there are of

| | Surgeons and Apothecaries. | | Physicians. |
|---|---|---|---|
| | 20 years of age and upwards. | Under 20 years of age. | Over 20 years of age. |
| Great Britain, . . . . | 17,006 | 1,451 | 1,476 |
| England, . . . . . . | 14,102 | 1,320 | 1,063 |
| Wales, . . . . . . . | 526 | 75 | 40 |
| Scotland, . . . . . . . | 2,237 | 248 | 464 |
| Isles in the British Seas. | 141 | 8 | 19 |

